# Cohort Profile: The National Institute for Health Research Health Informatics Collaborative: Hepatitis B Virus (NIHR HIC HBV) research dataset

**DOI:** 10.1093/ije/dyac127

**Published:** 2022-06-16

**Authors:** Tingyan Wang, David A Smith, Cori Campbell, Oliver Freeman, Zuzana Moysova, Theresa Noble, Kinga A Várnai, Steve Harris, Hizni Salih, Gail Roadknight, Stephanie Little, Ben Glampson, Luca Mercuri, Dimitri Papadimitriou, Christopher R Jones, Vince Taylor, Afzal Chaudhry, Hang Phan, Florina Borca, Josune Olza, Frazer Warricker, Luis Romão, David Ramlakhan, Louise English, Paul Klenerman, Monique Andersson, Jane Collier, Alexander J Stockdale, Stacy Todd, Karl McIntyre, Andrew Frankland, Eleni Nastouli, Salim I Khakoo, William Gelson, Graham S Cooke, Kerrie Woods, Jim Davies, Eleanor Barnes, Philippa C Matthews

**Affiliations:** NIHR Oxford Biomedical Research Centre, Oxford, UK; Nuffield Department of Medicine, University of Oxford, Oxford, UK; Nuffield Department of Medicine, University of Oxford, Oxford, UK; NIHR Health Informatics Collaborative, Oxford University Hospitals NHS Foundation Trust, Oxford, UK; NIHR Oxford Biomedical Research Centre, Oxford, UK; Nuffield Department of Medicine, University of Oxford, Oxford, UK; NIHR Oxford Biomedical Research Centre, Oxford, UK; Nuffield Department of Population Health, University of Oxford, Oxford, UK; NIHR Oxford Biomedical Research Centre, Oxford, UK; NIHR Health Informatics Collaborative, Oxford University Hospitals NHS Foundation Trust, Oxford, UK; NIHR Oxford Biomedical Research Centre, Oxford, UK; NIHR Health Informatics Collaborative, Oxford University Hospitals NHS Foundation Trust, Oxford, UK; NIHR Oxford Biomedical Research Centre, Oxford, UK; NIHR Health Informatics Collaborative, Oxford University Hospitals NHS Foundation Trust, Oxford, UK; NIHR Oxford Biomedical Research Centre, Oxford, UK; Department of Computer Science, University of Oxford, Oxford, UK; NIHR Oxford Biomedical Research Centre, Oxford, UK; NIHR Oxford Biomedical Research Centre, Oxford, UK; NIHR Oxford Biomedical Research Centre, Oxford, UK; NIHR Health Informatics Collaborative, Imperial College Healthcare NHS Trust, London, UK; NIHR Imperial Biomedical Research Centre, London, UK; NIHR Health Informatics Collaborative, Imperial College Healthcare NHS Trust, London, UK; NIHR Imperial Biomedical Research Centre, London, UK; NIHR Health Informatics Collaborative, Imperial College Healthcare NHS Trust, London, UK; NIHR Imperial Biomedical Research Centre, London, UK; NIHR Imperial Biomedical Research Centre, London, UK; Department of Infectious Disease, Imperial College London, London, UK; Department of Oncology, Cambridge University Hospitals NHS Foundation Trust, Cambridge, UK; Department of Nephrology, Cambridge University Hospitals NHS Foundation Trust, Cambridge, UK; NIHR Southampton Biomedical Research Centre, University Hospital Southampton NHS Foundation Trust, Southampton, UK; Clinical Informatics Research Unit, Faculty of Medicine, University of Southampton, Southampton, UK; NIHR Southampton Biomedical Research Centre, University Hospital Southampton NHS Foundation Trust, Southampton, UK; Clinical Informatics Research Unit, Faculty of Medicine, University of Southampton, Southampton, UK; NIHR Southampton Biomedical Research Centre, University Hospital Southampton NHS Foundation Trust, Southampton, UK; NIHR Southampton Biomedical Research Centre, University Hospital Southampton NHS Foundation Trust, Southampton, UK; NIHR University College London Hospitals Biomedical Research Centre, London, UK; NIHR University College London Hospitals Biomedical Research Centre, London, UK; NIHR University College London Hospitals Biomedical Research Centre, London, UK; Nuffield Department of Medicine, University of Oxford, Oxford, UK; Department of Infectious Diseases and Microbiology, Oxford University Hospitals NHS Foundation Trust, Oxford, UK; Department of Infectious Diseases and Microbiology, Oxford University Hospitals NHS Foundation Trust, Oxford, UK; Department of Hepatology, Oxford University Hospitals NHS Foundation Trust, Oxford, UK; Institute of Infection, Veterinary and Ecological Sciences, University of Liverpool, Liverpool, UK; Tropical Infectious Diseases Unit, Royal Liverpool Hospital, Liverpool University Hospitals NHS Trust, Liverpool, UK; Institute of Infection, Veterinary and Ecological Sciences, University of Liverpool, Liverpool, UK; Liverpool Clinical Laboratories, Liverpool University Hospitals NHS Trust, Liverpool, UK; Liverpool Clinical Laboratories, Liverpool University Hospitals NHS Trust, Liverpool, UK; Department of Clinical Virology, University College London Hospital, London, UK; Department of Infection, Immunity and Inflammation, University College London Great Ormond Street Institute of Child Health, London, UK; School of Clinical and Experimental Sciences, Faculty of Medicine, University of Southampton, Southampton, UK; Cambridge Liver Unit, Cambridge University Hospitals NHS Foundation Trust, Cambridge, UK; NIHR Health Informatics Collaborative, Imperial College Healthcare NHS Trust, London, UK; NIHR Imperial Biomedical Research Centre, London, UK; Faculty of Medicine, Department of Infectious Disease, Imperial College London, London, UK; NIHR Oxford Biomedical Research Centre, Oxford, UK; NIHR Health Informatics Collaborative, Oxford University Hospitals NHS Foundation Trust, Oxford, UK; NIHR Oxford Biomedical Research Centre, Oxford, UK; Department of Computer Science, University of Oxford, Oxford, UK; Nuffield Department of Medicine, University of Oxford, Oxford, UK; NIHR Health Informatics Collaborative, Oxford University Hospitals NHS Foundation Trust, Oxford, UK; Nuffield Department of Medicine, University of Oxford, Oxford, UK; NIHR Health Informatics Collaborative, Oxford University Hospitals NHS Foundation Trust, Oxford, UK; Francis Crick Institute, London, UK; Division of Infection and Immunity, University College London, London, UK; Department of Infectious Diseases, University College London Hospital, London, UK

Key Features

- The National Institute for Health Research (NIHR) Health Informatics Collaborative (HIC) has established a cohort of individuals with chronic hepatitis B virus (HBV) infection in secondary care in the UK, providing a resource for translational research.
- The dataset comprises >6000 individuals (99% adults aged 18–88, 1% children aged 1–17) with diverse ethnicities (32% Asian, 23% Black, 30% White and 15% mixed or other ethnic groups) from six NHS Trusts across England, representing data collected between August 1994 and October 2021.
- The dataset is populated with routinely collected clinical data captured from electronic patient record (EPR) systems; follow-up frequency of each individual depends on clinical practice, with a median of 5.1 (IQR: 2.8–8.0) years.
- Data on demographics, laboratory tests, antiviral treatment, elastography scores, imaging/biopsy reports, death information and potential risk factors for liver disease have been collected.
- Over time, the cohort will continue to grow in size, average follow-up duration will increase and more NHS Trusts will participate.
- This dataset offers important opportunities for epidemiological studies and biomedical informatics research, as well as characterizing an HBV population for clinical trials, including external collaborations with industry.
- Collaborations are welcomed, further details are available at [https://hic.nihr.ac.uk]. Queries regarding data access should be directed to [orh-tr.nihrhic@nhs.net].

## Why was the cohort set up?

Chronic infection with hepatitis B virus (HBV) is a global health problem, resulting in an estimated ∼887 000 deaths worldwide in 2015.^1^ Unlike deaths from other infections such as tuberculosis, human immunodeficiency virus (HIV) or malaria, the number of viral hepatitis deaths [the majority of which are attributable to HBV and hepatitis C virus (HCV) infection] has increased since 1990.^2^ To advance towards international goals for eliminating viral hepatitis,^3^ it is important to accurately estimate the baseline burden, to develop and deliver interventions based on real-world data and to monitor progress towards targets at regional and national levels.^4^

As the prevalence of HBV infection is low across the UK overall, there are limited data describing population characteristics and disease burden.^5^^,^^6^ Chronic HBV (CHB) nevertheless presents a concern in certain populations, either as a result of increased prevalence and/or risk factors for the development of long-term liver disease (e.g. chronic coinfection with HIV^7^ or other hepatitis viruses,^8^^,^^9^ diabetes mellitus or metabolic syndrome,^10^^,^^11^ alcohol abuse,^11^ migrants from countries/regions with a high prevalence of HBV^12^^,^^13^). Chronic infection can lead to pathology which has a major impact on quality of life and life expectancy, including cirrhosis, end-stage-liver failure and hepatocellular carcinoma (HCC). Following the successes of direct-acting antiviral drugs for HCV treatment as well as potential cure strategies targeting the reservoir in HIV infection, the clinical and research communities have focused progressive attention on cure strategies for HBV. There is therefore a pressing need for national-level data collection to evaluate population characteristics, identify risk factors, assess treatment deployment, develop predictive models for outcomes and provide a foundation for clinical trials for HBV.

Leveraging existing clinical data is a cost-effective way to build a detailed description of HBV infection. Existing primary care datasets like Clinical Practice Research Datalink (CPRD)^6^ do not capture HBV data well, as surveillance and treatment are largely managed in secondary care. During the past decade, large amounts of routinely collected clinical data have been accumulated in electronic patient record (EPR) systems in the UK’s unified secondary care services. The National Institute for Health Research (NIHR) Health Informatics Collaborative (HIC) collaboration was established in 2014 to enable re-use of these ‘big’ data for translational research.^14^

The NIHR HIC viral hepatitis theme provides a framework for collection of data for HBV, HCV and hepatitis E virus (HEV) in secondary care across National Health Service (NHS) Trusts (distinct regional organizations, each a separate legal entity, responsible for provision and commissioning of health care) in England, UK. In this cohort profile, we specifically introduce the large prospective multicentre cohort for CHB established within this theme. The challenges that had to be overcome in order to share data included establishing a unified governance framework across separate organizations, variations in data entry practice, data definitions and clinical practice between sites, de-identification required for large amounts of important free-text data and different levels of expertise in clinical informatics in different sites.^14^

With funding from the NIHR HIC and local support by NIHR Biomedical Research Centres (BRCs) at participating sites, the dataset continues to expand over time, with additional NHS Trusts joining the NIHR HIC viral hepatitis theme, and existing members refining the quality and quantity of data submitted.

## Who is in the cohort?

### Locations and setting

**Figure 1:**
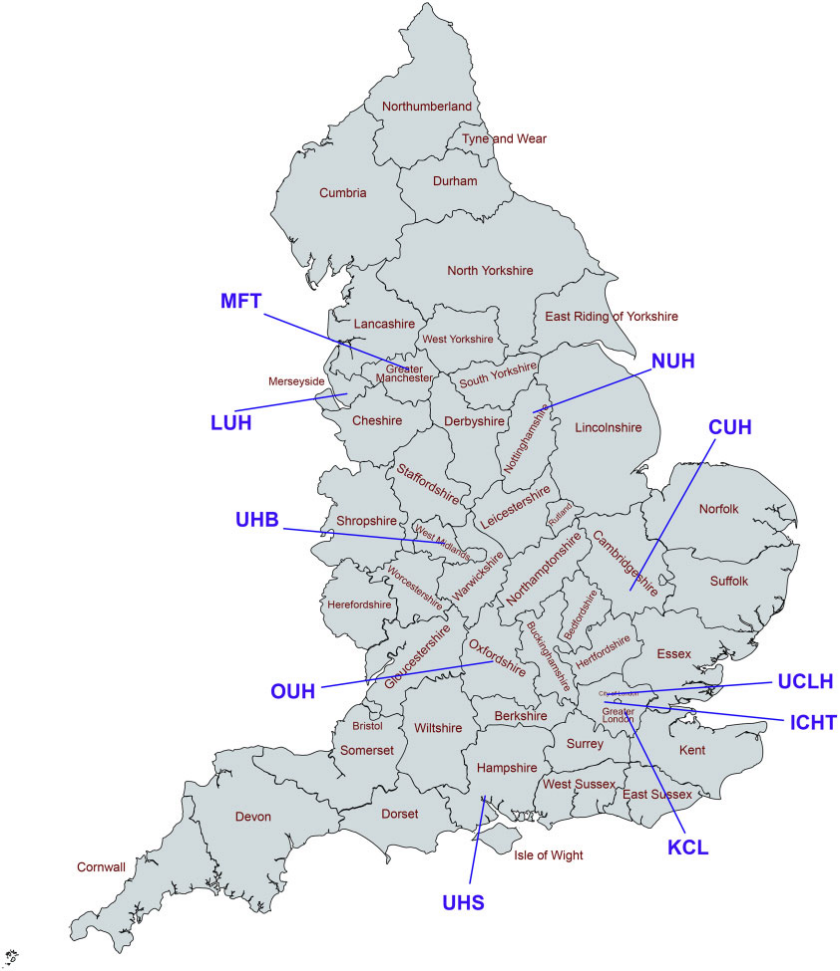
Locations of the 10 National Health Service (NHS) Trusts participating in the National Institute for Health Research (NIHR) Health Informatics Collaborative (HIC) viral hepatitis theme up to February 2022. CUH, Cambridge University Hospitals NHS Foundation Trust; ICHT, Imperial College Healthcare NHS Trust; KCL, King's College Hospital NHS Foundation Trust; LUH, Liverpool University Hospitals NHS Foundation Trust; MFT, Manchester University NHS Foundation Trust; NUH, Nottingham University Hospitals NHS Trust; OUH, Oxford University Hospitals NHS Foundation Trust; UCLH, University College London Hospitals NHS Foundation Trust; UHB, University Hospitals Birmingham NHS Foundation Trust; UHS, University Hospital Southampton NHS Foundation Trust. CUH, ICHT, KCL, OUH and UCLH were the five NHS Trusts initially included in the NIHR HIC viral hepatitis theme, with LUH, MFT, NUH, UHB and UHS joining more recently. The map was created using [https://www.mapchart.net/]

The NIHR HIC HBV cohort is a multisite dataset populated with anonymized routinely collected clinical data from individuals (including adults and children) with CHB attending secondary care services across the UK. Current data are from England, but the NIHR HIC provides opportunities to expand the dataset to represent other locations within the UK. The locations of the current 10 participating sites are shown in Figure 1, of which six have submitted data up to February 2022.

At each site, routinely collected clinical data are captured in local electronic systems. However, these systems were originally designed for local clinical services rather than for research purposes, so data entry practice and storage format are not unified. Different sites use different types of EPR system for clinical solutions (e.g. Cerner Millennium, Epic), and even when sites are using the same type of EPR system, the data record style and integration are locally customized. To overcome such challenges, we have developed an informatics infrastructure and established a comprehensive governance framework for collecting data between heterogeneous EPR environments, detailed previously.^14^ All the laboratory assays used at each site were undertaken on validated platforms in UK laboratories with clinical accreditation.

Data since the date of EPR system implementation are retrospectively captured, though historical data pre-dating the implementation are also included at some sites. Further data are added prospectively, with updates submitted on request and transferred to the theme central data repository. Thus, the start date (earliest available data) can vary by years between Trusts, due to different time lines of EPR system introduction, but the end date (latest available data) is mostly within the same calendar year.

The central data repository of the NIHR HIC HBV dataset is hosted by the theme lead centre (Oxford University Hospitals NHS Foundation Trust) under a governance framework that includes a data sharing agreement and terms on contractual responsibilities, confidentiality, intellectual property and publication.^14^ Data subjects (patients) are informed about the processing and data use via the Trusts’ Privacy Notices and public facing materials, and can opt out from having their data shared with this dataset via the National Data Opt-out. A scientific steering committee, made up of at least one representative from each participating site, meets regularly to review data collection, feedback progress on active projects, consider updates to the database and review all data requests.

### Data anonymization and data protection

Each participating Trust anonymizes their data by removing direct patient identifiers locally and assigning each unique patient a study identifier prior to transmitting data to the central data repository.^14^ This allows researchers to conduct analysis without the possibility of patient identification. To avoid submitting duplication of records to the lead centre, each Trust is responsible for locally maintaining a link between the patient’s local identifier and the anonymous study identifier used in the dataset.^14^ The central data repository is based on a secure data access platform and only authorized personnel are permitted to access data. To access the data for research purposes, a formal request with research proposals must be submitted to the theme scientific steering committee for review. When reporting or publishing data, information for small numbers (e.g. less than five) study subjects in special groups are suppressed to avoid the risk of individuals being identified, as per national guidelines on managing data protection risk.^15^^,^^16^

### Eligibility criteria

The inclusion criteria up to May 2021 were (i) individuals for whom data are recorded in the EPR systems; and (ii) individuals with CHB, defined by two positive hepatitis B surface antigen (HBsAg) tests and/or detectable HBV DNA at least 6 months apart (Supplementary Table S1, available as Supplementary data at *IJE* online). In June 2021, an update was agreed to relax the criteria for inclusion, such that a single positive HBsAg or HBV DNA test was considered sufficient. Although this potentially adds a small number of cases of acute infection, it renders many more cases of chronic infection eligible for data inclusion and thus provides a more complete picture of all HBV infections. The cases with acute infection can be subsequently excluded when analysis is performed on the dataset, if the study requires a stringent case definition of chronic infection. The exclusion criteria were: (i) patients without records of demographics or (ii) patients without mandated laboratory data (Supplementary Tables S2, S3, available as Supplementary data at *IJE* online) in the EPR systems (Supplementary Figure S1, available as Supplementary data at *IJE* online).

### Index date, baseline period and numbers of subjects

**Figure 2:**
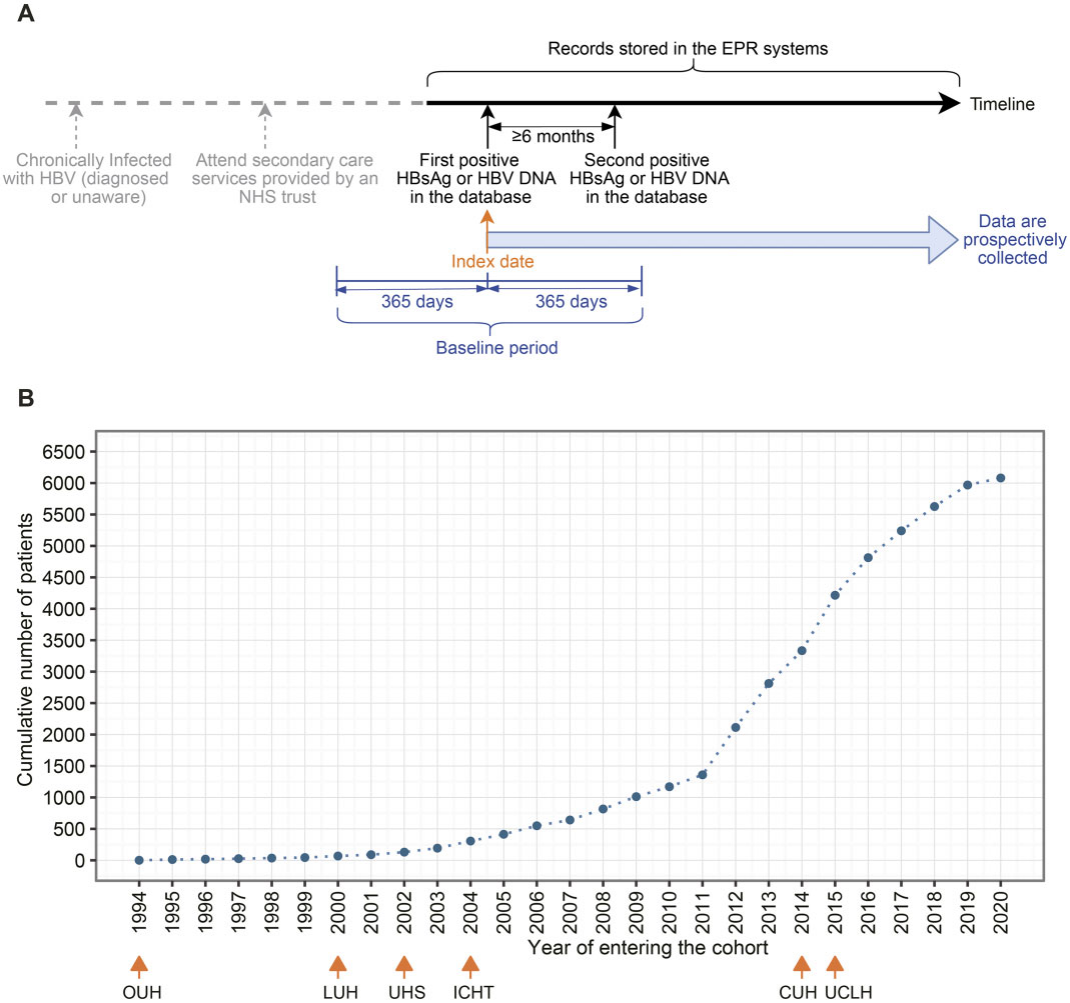
Index date and number of patients in the National Institute for Health Research (NIHR) Health Informatics Collaborative (HIC) hepatitis B virus dataset. (A) The time line and index date definition for an exemplar patient. (B) Cumulative numbers of chronic hepatitis B virus patients entering the cohort over time. In panel A, all data collected after the index date are added into the dataset, and those data before index date are also included if they are available in EPR systems. The second positive test could be within 365 days after the index date (within baseline period) or could be >365 days after the index date (outside of the baseline period). Note that the second positive test is not required for a contributing site to include patients for data submission after June 2021. In panel B, for the six sites which had submitted data, the earliest year of the included data at each site is marked. CUH, Cambridge University Hospitals NHS Foundation Trust; ICHT, Imperial College Healthcare NHS Trust; LUH, Liverpool University Hospitals NHS Foundation Trust; OUH, Oxford University Hospitals NHS Foundation Trust; UCLH, University College London Hospitals NHS Foundation Trust; UHS, University Hospital Southampton NHS Foundation Trust; EPR, electronic patient record; HBsAg, hepatitis B surface antigen; HBV, hepatitis B virus; DNA, deoxyribonucleic acid; NHS, National Health Service

For each individual, we defined the first episode of positive HBsAg or HBV DNA recorded in the EPR system as their ‘index date’ (Figure 2A). A baseline period was defined as 365 days within the index date. For some patients, the index date may be later than the time when they were clinically diagnosed with CHB due to geographical migration across regions/countries. We are unable to capture the retrospective data that might be stored in a different Trust for patients even when data from this Trust are added into the dataset, as it is not possible to map patients between Trusts within the dataset due to anonymization.

At the most recent update in February 2022, the NIHR HIC HBV dataset consists of 6080 CHB patients, with index dates between August 1994 and December 2020. Cumulative numbers of cases in the cohort over time are presented in Figure 2B. Individuals with age <18 years (*n* = 89) are not described in the remaining text but they are included in the dataset when they reach 18 years of age.

## How often have they been followed up?

Individuals were followed from the index date until they died or were lost to follow-up (defined as no new records within 24 months of the most recent data update). Patients lost to follow-up, who are subsequently re-enrolled into care in their original Trust, would be included back into the dataset as the same participant; whereas if later they move into another Trust which is contributing to this dataset, they would appear as a new participant once they have hepatitis B serology done. The follow-up frequency of each individual is variable (influenced by clinical requirements and patient preference), and is subject to influence by other factors, including disruptions to clinical services caused by the COVID-19 pandemic since early 2020.

### Follow-up duration and frequency, and availability of longitudinal data

Currently, median follow-up duration of the adult patients in this dataset has been 5.1 (IQR: 2.8–8.0) years; 5.46% (327/5991) of patients died during follow-up, with 9.3 (95% CI: 8.3–10.4) deaths per 1000 person-years, similar to the mortality rate reported by an Asian study of CHB patients with similar age profile.^17^ The demographics, follow-up duration and coinfection characteristics of adults who died (*n *= 327) or were lost to follow-up (*n* = 1301), compared with those who are active (*n *= 4363) in the cohort, are presented in Table 1.

**Table 1:** Demographics, follow up duration and coinfection characteristics of adults with chronic hepatitis B virus who died or were lost to follow-up vs who are active in the cohort

Parameter	Active	Died	Lost to follow-up	*P*-value	*P*-value lost to follow-up vs active
	*n* = 4363	*n *= 327	*n *= 1301	died vs active	
Follow-up duration, years, median [IQR]	5.6 [3.8, 8.4]	3.9 [1.9, 7.2]	2.9 [1.5, 5.2]	<0.0001	<0.0001
Gender, male, *n* (%)	2343 (53.7)	256 (78.3)	686 (52.7)	<0.0001	0.5580
Age, years, median [IQR]	40.0 [32.0, 50.0]	58.0 [47.0, 68.0]	35.0 [29.0, 44.0]	<0.0001	<0.0001
Age group, years, *n* (%)				<0.0001	<0.0001
18–24	212 (4.9)	<5 (-)	126 (9.7)		
25–34	1233 (28.3)	14 (4.3)	496 (38.1)		
35–44	1304 (29.9)	47 (14.4)	374 (28.7)		
45–54	894 (20.5)	67 (20.5)	190 (14.6)		
55–64	490 (11.2)	85 (26.0)	67 (5.1)		
65–74	184 (4.2)	74 (22.6)	39 (3.0)		
>=75	46 (1.1)	38 (11.6)	9 (0.7)		
Ethnic groups, *n* (%)				<0.0001	<0.0001
Asian	1206 (27.6)	77 (23.5)	240 (18.4)		
Black	839 (19.2)	58 (17.7)	213 (16.4)		
Mixed	117 (2.7)	<5 (-)	39 (3.0)		
White	1021 (23.4)	126 (38.5)	276 (21.2)		
Other	432 (9.9)	27 (8.3)	122 (9.4)		
Not stated	748 (17.1)	36 (11.0)	411 (31.6)		
HCV coinfection, *n* (%)	149 (3.4)	49 (15.0)	31 (2.4)	<0.0001	0.0762
HDV coinfection, *n* (%)	51 (1.2)	<5 (-)	8 (0.6)	0.8867	0.1160
Past/acute HEV infection, *n* (%)	33 (0.8)	5 (1.5)	<5 (-)	0.2366	0.0256
HIV coinfection, *n* (%)	78 (1.8)	6 (1.8)	13 (1.0)	1	0.0629

Square brackets for IQR, round brackets for % of column total.

IQR, interquartile range; HCV, hepatitis C virus; HDV, hepatitis D virus; HEV, hepatitis E virus; HIV, human immunodeficiency virus.

**Figure 3:**
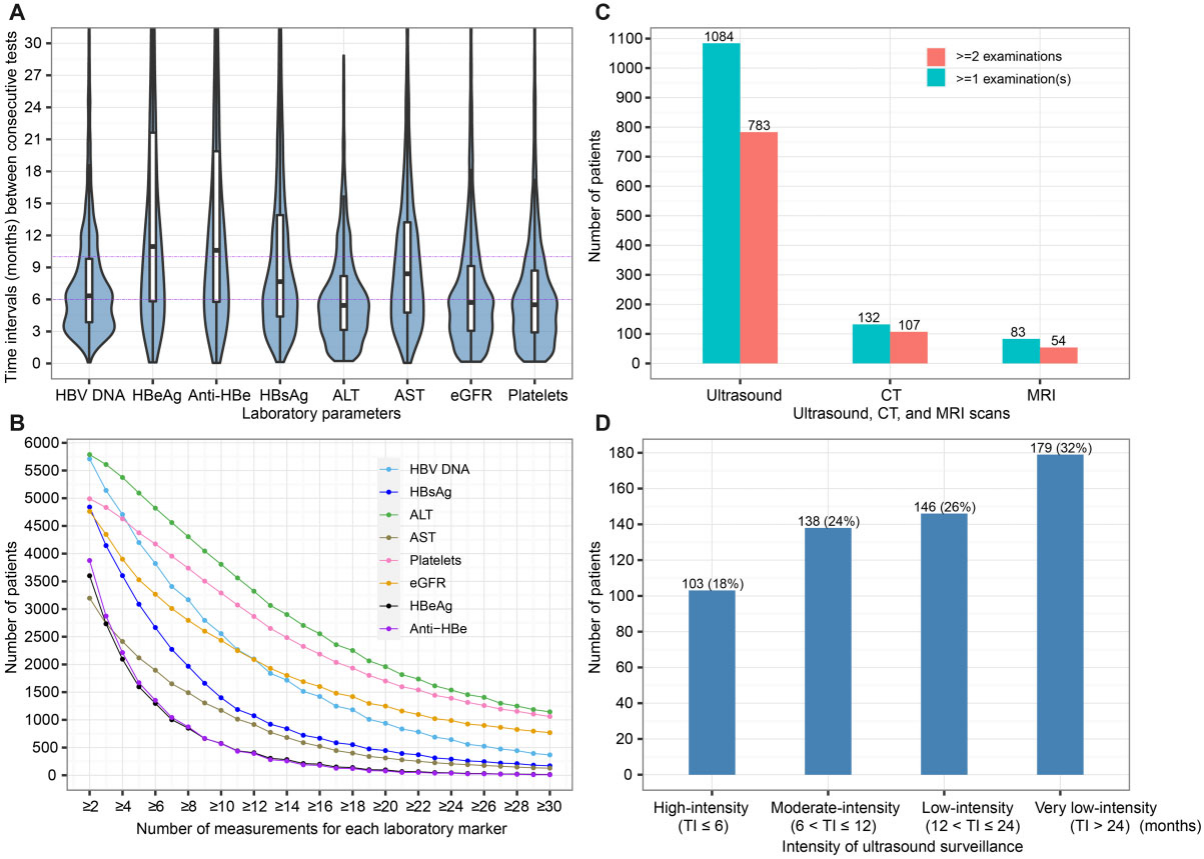
Follow-up frequency and longitudinal data availability. (A) Time intervals (months) between two consecutive tests within patients of hepatitis B virus serological and virological biomarkers, liver biochemistry parameters and renal function markers. (B) Numbers of patients who had longitudinal data (i.e. ≥ two measurements) of laboratory markers [hepatitis B virus deoxyribonucleic acid (HBV DNA), hepatitis B surface antigen (HBsAg), hepatitis B e antigen (HBeAg), antibody to HBeAg (Anti-HBe), alanine aminotransferase (ALT), aspartate aminotransferase (AST), estimated glomerular filtration rate (eGFR), platelets] during the follow-up period. (C) Patients with ultrasound, computed tomography (CT), or magnetic resonance imaging (MRI) surveillance data with various numbers of examinations. (D) Patients with differing intensity of ultrasound surveillance for those who had two or more ultrasound scans. In panel A, mean value of time intervals between every two consecutive tests for each patient was calculated, then the violin plot and boxplot were drawn based on these mean values with outliers (the observations below the 1st percentile and the observations above the 99th percentile) removed. Boxplots indicate the median and quartiles with whiskers reaching up to 1.5 times the interquartile range. The violin plot outlines illustrate kernel probability density, i.e. the width of the blue shaded area represents the proportion of the data located there. Data beyond 30 months were not shown in the plots. In panel B, the x-axis indicates the number of measurements for patients who had longitudinal data (i.e., two or more measurements) on a test. TI, time interval

Laboratory parameters such as HBV DNA, alanine aminotransferase (ALT), platelets and estimated glomerular filtration rate (eGFR) were assessed with a median interval of ∼6 months, and were more frequently measured than HBsAg, hepatitis B e antigen (HBeAg), antibody to HBeAg (anti-HBe) and aspartate aminotransferase (AST), assessed with a median interval of 8–10 months (Figure 3A). Most patients (80%–97%) had ≥ 2 ALT, HBV DNA, HBsAg, platelets and eGFR measurements, and a lower proportion (60%, 65%, 53%, respectively) had ≥2 HBeAg, anti-HBe and AST measurements (Figure 3B), reflecting differences in various patient populations or clinical practice between sites.

In line with clinical guidelines, ultrasound is routinely used for surveillance; CT and MRI scans are less frequently used (typically only if concerns are raised by other imaging, laboratory or clinical features). Three sites (contributing data for 1882 adults with CHB) have submitted imaging reports to date. During follow-up, 1084/1882 (57.6%) and 783/1882 (41.6%) patients had one or more and two or more ultrasound examination(s), respectively (Figure 3C). For those with two or more ultrasound examinations, 18% were on high-intensity surveillance (≤6 months), 24% on moderate-intensity surveillance (>6–12 months), 26% on low-intensity surveillance (>12–24 months) and 32% on surveillance with intervals >24 months (Figure 3D).

## What has been measured?

### Data model

A standardized data model, used by all collaborating sites for data mapping, extraction and submission, has been designed and released in the Mauro Data Mapper (used as the NIHR HIC’s metadata catalogue; see: https://modelcatalogue.cs.ox.ac.uk/nihr-hic/#/home). An overview of data classes and elements defined in the data model is provided in Supplementary Table S2 and detailed definitions are provided in the Supplementary XML Schema Definition (XSD) file (available as Supplementary data at *IJE* online). Individual-level data on demographics, death information, laboratory tests, antiviral treatment, elastography scores, imaging reports, liver biopsy reports and potential risk factors for liver disease are collected. Additionally, three new data classes (diagnostic codes, deprivation scores and health care utilisation) have been added into the new version of the data model (v2.1.0, released in December 2021) (Supplementary Table S2). Definitions for data elements, such as ethnicity, country of birth and cause of death, were taken from the standardized NHS Data Dictionary [https://www.datadictionary.nhs.uk/]. Death records are collected from the Office for National Statistics through NHS Digital’s Spine portal, and date of death is checked for each patient before data are submitted. All data classes are linked to produce a complete record for each unique patient.

### Data inference

One principle of the designed data model is to collect source data as they appear in EPR systems and to allow researchers to infer information of interest using raw data collected. All the inferred fields included in the data model are presented in the Supplementary XML schema definition (XSD) file. Here, we used the inferred variables coinfection exposures and liver disease severity.

Chronic viral coinfections (HIV, HCV, HDV), and acute infection or past exposure to HEV, were identified from laboratory tests (Supplementary Table S3). Liver fibrosis and cirrhosis were characterised based on Ishak or METAVIR scores from biopsy reports^18^ or on liver stiffness measurements from transient elastography (FibroScan) if available; otherwise, we used AST to platelet ratio index (APRI)^19^ or Fibrosis-4 (FIB-4) scores^20^ (Supplementary Figure S2, available as Supplementary data at *IJE* online). We used pre-defined thresholds for significant/advanced fibrosis and cirrhosis: 1.5 and 2.0 for APRI score, respectively;^19^ 3.25 and 3.6 for FIB-4 score, respectively.^20^^,^^21^ Decompensation and HCC information was retrieved from clinical and imaging reports if available.

## What has it found?

At baseline, for adults (*n* = 5991), the median age was 39 years (IQR: 32–50) and 55% were male; 4796 had ethnicity recorded, among whom 32% were Asian, 23% were Black, 30% were White and the remaining 15% were mixed or other ethnic groups (Supplementary Table S4, available as Supplementary data at *IJE* online).

This present cohort (as per updates up to February 2022) comprises CHB patients of diverse ethnicities from six secondary care NHS Trusts across England, mostly representing adults in middle life. The proportion of patients receiving antiviral treatment varies by gender, age and ethnicity, which warrants further investigation. A large majority of patients in this cohort had longitudinal measurements of relevant laboratory parameters, providing promising opportunities for longitudinal analyses.

We have already undertaken studies using this framework, with more in process. During the COVID-19 pandemic, we have investigated service disruptions, revealing that reduction in rates of surveillance closely track COVID-19 incidence and periods of population lock-down.^22^ Using this dataset, we have reported a bimodal viral load distribution^23^ and found evidence of a virological set point in untreated patients.^23^^,^^24^ In a comparison of tenofovir disoproxil fumarate (TDF)-treated vs untreated patients, we reported variable ethnicity distributions across the two groups and some evidence for liver fibrosis progression in the untreated group, highlighting a need for further evidence for expanded treatment.^24^ A study of HBsAg and HBeAg clearance dynamics demonstrated that these markers may contribute to prognostication and patient-stratified care and provide a foundation for advancing insights into mechanisms of disease control.^25^ The list of publications is available in [https://hic.nihr.ac.uk] with ongoing/planned studies presented in Table 2.

**Table 2:** Ongoing and planned studies using National Institute for Health Research (NIHR) Health Informatics Collaborative (HIC) Hepatitis B virus dataset

Studies	Goals
HBV treatment eligibility and coverage	To assess HBV treatment eligibility and coverage of patients in this cohort, as well as to investigate which NICE treatment criteria and patient characteristics are associated with odds of receiving or not receiving treatment
Early prediction of HBsAg loss	To apply advanced machine learning techniques to predict HBsAg loss and determine key factors associated with this endpoint
HCC incidence and associated factors	To investigate factors affecting the incidence of HCC in patients with viral hepatitis
HCC identification	To develop a natural language processing (NLP) pipeline to automatically identify HCC from imaging reports
Metabolic factors and CHB outcomes	To explore the association between metabolic risk factors and CHB outcomes
HBV treatment failure risk prediction	To investigate virological suppression patterns and predict the risk of not suppressing or rebound viraemia in chronic HBV patients with antiviral treatment

This list is not exhaustive, and more studies will be added over time.

HBV, hepatitis B virus; NICE, National Institute for Health and Care Excellence; HBsAg, hepatitis B surface antigen; HCC, hepatocellular carcinoma; CHB, chronic hepatitis B virus.

## What are the main strengths and weaknesses?

As the biggest dataset reflecting CHB in secondary care in England, and growing year on year with improving quality, the NIHR HIC HBV dataset is an invaluable resource for answering diverse questions, supporting collaborations, refining approaches for care stratification and treatment and influencing policy for health interventions. As the HBV field moves towards new therapeutics, with a quest for cure strategies, clear information about the characteristics of HBV infection in different settings will be essential to underpin the design and implementation of clinical trials and ultimately to inform equitable access to treatment.

The strengths of NIHR HIC HBV derive from the broad interdisciplinary and cross-site collaboration among clinicians (including hepatology, infectious disease and microbiology specialists), informaticians, project managers and data managers/analysts, representing the NHS Trusts, the NIHR BRCs and the affiliated universities actively participating in the research. Each NHS Trust publishes regularly updated Patient and Public Involvement strategies and engages with patients and the public about the research supported using Trust resources on a regular basis.

The multisite approach integrates CHB data for a broad cross-section of populations from secondary care, and produces comprehensive records on a large scale, with an automatic data validation process. Longitudinal clinical data are particularly important for informing treatment and stratification. Data collection is continuing, with the sample size growing, collection of more parameters being completed, average follow-up increasing and expansion to include more NHS Trusts across the UK. The diversity and statistical power of the dataset will therefore be enhanced for future analyses, providing robust and reliable results despite heterogeneous intrinsic characteristics that exist in patients from different sources.

We recognize limitations which can influence data quality and completeness. Although assays are performed on validated platforms, methods of laboratory tests vary by site or period, e.g. different approaches are used for HBsAg quantification and variable equations are used for eGFR calculation. Therefore, data may need calibration or transformation before analyses, and differences must be flagged before data comparison across sites. As different Trusts prioritize different tests, various levels of missingness exist in liver biochemistry like AST and in serology markers such as HBeAg and anti-HBe. However, these data can influence planning and improving the standard and consistency of clinical care, as well as improving access to new treatments as these become available. Data at some sites are not currently linked to national registries/sources such as the Office for National Statistics death registrations. Additionally, free-text imaging and liver biopsy reports are not systematically available, as anonymization processes that are novel to some sites must be performed before data can be shared. Meanwhile, some data points are difficult to capture from EPR systems, such as treatment records stored in local pharmacy systems, elastography scores recorded in inconsistent and inaccessible formats and self-reported alcohol data not consistently recorded. Although data noise is a common limitation accompanying use of routinely collected clinical data, findings will become more robust as larger study populations are assimilated, electronic systems become better at data capture and the data model is further refined.

Our original inclusion criteria required two episodes of positive HBsAg and/or HBV DNA tests ≥ 6 months apart, which might result in some cases with missing data being excluded. The relaxation of the inclusion criteria from June 2021 to one positive HBsAg and/or HBV DNA test will provide a wider population available for investigation, while still allowing researchers to apply their own criteria to narrow down the population to include only the more stringent diagnosis of CHB if required for a particular question. Additionally, many individuals with HBV infection are not diagnosed or not receiving clinical care, and thus not represented in secondary care datasets. These individuals may include a disproportionate number in vulnerable groups, including migrants^26^ (and perhaps specifically non-English speakers), people who inject drugs^27^ and those in prison or detention centres.^28^

Although comparable HBV datasets are more available in other countries, such as China and the USA,^29–31^ there are scarce comprehensive data of HBV in the UK except for data reported from certain populations^32–34^ or the primary care population.^5^^,^^6^ We believe this secondary care cohort can start to fill evidence gaps, especially by collating laboratory, imaging and treatment data which are not currently well captured in primary care.

As an exemplar case, this cohort profile not only highlights the potential utility of a CHB cohort, but also demonstrates that routine clinical data are a valuable resource for translational research. Our use of data during the COVID-19 pandemic^35^ highlights how the resource can be quickly adapted to address new questions as they arise.

## Can I get hold of the data? Where can I find out more?

Any potential collaborations are welcomed, and data may be made available to researchers on request following positive review by the steering committee. Further details are available at [https://hic.nihr.ac.uk]. Queries regarding data access and more information about the dataset can be sent to Prof. Eleanor Barnes [ellie.barnes@ndm.ox.ac.uk] or directed to [orh-tr.nihrhic@nhs.net].

## Ethics approval

The research database for the NIHR HIC viral hepatitis theme was approved by South Central—Oxford C Research Ethics Committee (REF Number: 21/SC/0060). All methods for data collection, transmission and management for the NIHR HIC HBV cohort were carried out in accordance with relevant guidelines and regulations. The requirement for written informed consent was waived by South Central—Oxford C Research Ethics Committee, because data have been anonymized before transmission to the theme central data repository.

## Supplementary Material

dyac127_Supplementary_Data

## Data Availability

See Can I get hold of the data? above.
