# Supplementary material for: Cohort Profile: The National Institute for Health Research Health Informatics Collaborative: Hepatitis B Virus (NIHR HIC HBV) research dataset
Source: Int J Epidemiol. 2022 Jun 16;52(1):e27–37. doi: 10.1093/ije/dyac127 (PMC9908046; doi:10.1093/ije/dyac127)
Supplement: dyac127_Supplementary_Data [file dyac127_supplementary_data.zip › dyac127_Supplementary_Data/ije-2021-10-1519-File008.docx]

**Author contributions**

EB, PCM, GSC, WG, EN, SIK, KW, and JD contributed to the conception or design of the work. EB, PCM, JD, KW, GSC, EN, WG, and SIK directed the study’s implementation. TW, DAS, CC, OF, ZM, TN, SH, HS, KAV, GR, SL, BG, LM, DP, CRJ, VT, AC, HP, FB, JO, FW, LR, DR, LE, PK, MA, JC, AJS, ST, KM, AF contributed to the methodology development and the acquisition, processing, interpretation, or management of study data. TW, DAS, CC designed the analytical strategy and TW conducted the analysis, supervised by EB and PCM. PCM, EB, WG, EN, GSC, SIK, PK, MA, and JC helped interpret the findings. TW, PCM, EB, DAS, CC, and KAV contributed to the original draft. All authors reviewed and revised the manuscript critically and approved the final version for publication.
